# Empirical Relative Biological Effectiveness (RBE) for Mandible Osteoradionecrosis (ORN) in Head and Neck Cancer Patients Treated With Pencil-Beam-Scanning Proton Therapy (PBSPT): A Retrospective, Case-Matched Cohort Study

**DOI:** 10.3389/fonc.2022.843175

**Published:** 2022-03-03

**Authors:** Yunze Yang, Olivia M. Muller, Satomi Shiraishi, Matthew Harper, Adam C. Amundson, William W. Wong, Lisa A. McGee, Jean-Claude M. Rwigema, Steven E. Schild, Martin Bues, Mirek Fatyga, Justin D. Anderson, Samir H. Patel, Robert L. Foote, Wei Liu

**Affiliations:** ^1^Department of Radiation Oncology, Mayo Clinic Arizona, Phoenix, AZ, United States; ^2^Department of Dental Specialties, Mayo Clinic Rochester, Rochester, MN, United States; ^3^Department of Radiation Oncology, Mayo Clinic Rochester, Rochester, MN, United States; ^4^School of Dentistry, West Virginia University, Morgantown, WV, United States

**Keywords:** relative biological effectiveness, mandibular osteoradionecrosis, linear energy transfer (LET), pencil beam scanning proton therapy (PBSPT), head and neck (H&N) cancer, volume modulated arc-therapy (VMAT), dose LET volume histogram (DLVH)

## Abstract

**Purpose:**

To retrospectively investigate empirical relative biological effectiveness (RBE) for mandible osteoradionecrosis (ORN) in head and neck (H&N) cancer patients treated with pencil-beam-scanning proton therapy (PBSPT).

**Methods:**

We included 1,266 H&N cancer patients, of which, 931 patients were treated with volumetric-modulated arc therapy (VMAT) and 335 were treated with PBSPT. Among them, 26 VMAT and 9 PBSPT patients experienced mandible ORN (ORN group), while all others were included in the control group. To minimize the impact of the possible imbalance in clinical factors between VMAT and PBSPT patients in the dosimetric comparison between these two modalities and the resulting RBE quantification, we formed a 1:1 case-matched patient cohort (335 VMAT patients and 335 PBSPT patients including both the ORN and control groups) using the greedy nearest neighbor matching of propensity scores. Mandible dosimetric metrics were extracted from the case-matched patient cohort and statistically tested to evaluate the association with mandibular ORN to derive dose volume constraints (DVCs) for VMAT and PBSPT, respectively. We sought the equivalent constraint doses for VMAT so that the critical volumes of VMAT were equal to those of PBSPT at different physical doses. Empirical RBEs of PBSPT for ORN were obtained by calculating the ratio between the derived equivalent constraint doses and physical doses of PBSPT. Bootstrapping was further used to get the confidence intervals.

**Results:**

Clinical variables of age, gender, tumor stage, prescription dose, chemotherapy, hypertension or diabetes, dental extraction, smoking history, or current smoker were not statistically related to the incidence of ORN in the overall patient cohort. Smoking history was found to be significantly associated with the ORN incidence in PBSPT patients only. V40Gy[RBE], V50Gy[RBE], and V60Gy[RBE] were statistically different (*p*<0.05) between the ORN and control group for VMAT and PBSPT. Empirical RBEs of 1.58(95%CI: 1.34-1.64), 1.34(95%CI: 1.23-1.40), and 1.24(95%: 1.15-1.26) were obtained for proton dose at 40 Gy[RBE=1.1], 50 Gy[RBE=1.1] and 60 Gy[RBE=1.1], respectively.

**Conclusions:**

Our study suggested that RBEs were larger than 1.1 at moderate doses (between 40 and 60 Gy[RBE=1.1]) with high LET for mandible ORN. RBEs are underestimated in current clinical practice in PBSPT. The derived DVCs can be used for PBSPT plan evaluation and optimization to minimize the incidence rate of mandible ORN.

## Introduction

Radiotherapy (RT) is a standard treatment option for head and neck (H&N) cancer. Adverse events are frequent after H&N cancer RT as there are a large number of adjacent organs-at-risk (OARs), resulting in significant increase in need for supportive care and subsequent decreased quality of life (1–3). Osteoradionecrosis (ORN) is one of the most severe adverse events for H&N cancer treatment.

Volumetric-modulated arc therapy (VMAT) and pencil-beam-scanning proton therapy (PBSPT) are two advanced modalities for external beam radiation therapy. VMAT is an advanced form of intensity-modulated photon-based RT (IMRT) that can deliver a precisely sculpted dose distribution using a single or multiple arcs (4). Comparatively, PBSPT is the most advanced generation of proton therapy. Because protons have a finite range (e.g., Bragg Peak) and no dose exists beyond Bragg Peaks, proton therapy provides more conformal target coverage while sparing adjacent OARs (5).

Despite the dosimetric benefits, PBSPT poses many challenges. Other than plan robustness (6–18), relative biological effectiveness (RBE) is a major issue (19, 20). In contrast to VMAT, protons impart most of their energy over a short distance, and thus induce high linear energy transfer (LET) near the distal end of the Bragg Peak. Hence, the biological dose of PBSPT is determined by both physical dose and LET (and possibly other factors) (20, 21). In clinical practice, a fixed RBE of 1.1 is used to describe the higher biological damaging effect of protons compared to photons. Various studies on *in vitro* cell experiments show that RBE increases with elevated LET (19, 22), while clinical outcome data is less clear regarding the impact of LET on RBE. Bahn et al. (23) suggested an RBE of 1.20 for LET of 2 keV/µm and 1.50 for LET of 5 keV/µm for the brain using a two-level normal tissue complication probability (NTCP) model based on a probability origin hypothesis. Recently, by comparing tolerance doses between the adverse event group and the control group, Zhang et al. (24) revealed an increased RBE for brain necrosis with RBE of 1.18 at a dose of 64.4 Gy[RBE=1.1] in passive scattering proton therapy. Unfortunately, the empirical RBE relationships to both dose and LET are still unclear.

In this study, we investigated the incidence of mandibular ORN based on patient outcomes of H&N cancer patients treated with PBSPT and VMAT at our institutions. Dose volume constraints (DVCs) for mandible ORN were obtained respectively for VMAT and PBSPT by comparing patients with (ORN group) and without ORN (control group). We derived empirical RBEs of PBSPT by calculating the ratios of the corresponding equivalent constraint doses and physical doses of PBSPT assuming the same critical volumes of the DVCs between PBSPT and VMAT. We described the relationship between LET and RBE using a recently developed concept of dose-LET volume histogram (DLVH) (25). To the best of our knowledge, this work represents one of the first comprehensive studies to define empirical RBEs for mandible ORN in patients treated with PBSPT based on patient outcomes.

## Methods

### Patient Cohort

The proposed studies are applicable to all newly diagnosed H&N cancer patients consecutively treated at Mayo Clinic Rochester and Arizona between April 2013 and August 2019 (>2 years’ follow-up) with curative intent definitive chemoradiation therapy (VMAT or PBSPT) regardless of gender, age, minority status, vulnerable population status, and weight with a confirmed histologic diagnosis. The data was limited to (1): the patients treated with fractionation sizes of 120 cGy[RBE] to 220 cGy[RBE] per fraction (2); the patients treated with the prescription dose of at least 60 Gy[RBE] to the high-risk tumor target for both modalities; and (3) the patients with re-treatment only if the dose to the mandible was negligible from the re-treatment plan or if patients had already developed ORN before re-treatment. In total we got 1,266 patients. Among them, 931 were treated with VMAT and 335 were treated with PBSPT. Patients were non-intentionally selected by any clinical factors for either treatment modality. Note that we used Gy[RBE] to present doses for both VMAT and PBSPT if the RBE value was not specified in the bracket. For PBSPT, a fixed value of RBE=1.1 was assumed following current clinical practice (*i.e.* 60 Gy[RBE=1.1]). For VMAT, Gy[RBE] represented the physical dose (Gy) (*i.e.* 60 Gy[RBE=1.0]).

Most of the ORN cases occurred in patients with a primary tumor arising within the oral cavity or oropharynx or with unknown primary metastatic to nodes, in which case the oropharynx was treated (VMAT: 530 vs. PBSPT: 161). All patient data were stored in our institutional patient outcomes database (26). Demographic (age, gender) and related clinical information (prescription dose, tumor stage, concurrent chemotherapy, hypertension, diabetes, dental extraction, smoking history (former or current smoker) and current smoker) were extracted (**Table 1**). This study was approved by our institutional review board (IRB).

**Table 1:** Characteristics for patient cohort from VMAT and PBSPT with and without ORN.

	Total	Photon		Proton	
		ORN	Ctr^a^	ORN	Ctr^a^
**Age**					
Median (range)	62 (11–93)	58 (46–77)	61 (14–93)	60 (46–83)	65 (11–91)
**Gender** [# of patients (% of patients)]					
Female	327 (25.8)	6 (23.1)	244 (27.0)	1 (11.1)	76 (23.3)
Male	939 (74.2)	20 (76.9)	661 (73.0)	8 (88.9)	250 (76.7)
**Tumor Stage** [# of patients (% of patients)]					
Stage I	130 (10.3)	2 (8.0)	82 (9.1)	2 (22.2)	44 (13.5)
Stage II	151 (11.9)	2 (8.0)	102 (11.3)	0 (0.0)	47 (14.4)
Stage III	182 (14.4)	4 (16.0)	137 (15.1)	2 (22.2)	39 (12.0)
Stage IV	669 (52.8)	16 (61.5)	512 (56.6)	4 (44.4)	137 (42.0)
Stage X (undefined)	134 (10.6)	2 (8.0)	72 (8.0)	1 (11.1)	59 (18.1)
Concurrent Chemotherapy^b^ [# of patients (% of patients)]					
w/concurrent chemotherapy	593 (58.0)	17 (70.8)	459 (58.6)	4 (66.7)	113 (54.1)
Hypertension^b^ [# of patients (% of patients)]					
w/hypertension	513 (50.2)	13 (54.2)	418 (53.3)	2 (33.3)	80 (38.3)
Diabetes^b^ [# of patients (% of patients)]					
w/diabetes	146 (14.3)	2 (8.3)	116 (14.8)	0 (0.0)	28 (13.4)
Dental Extraction^b^ [# of patients (% of patients)]					
w/dental extraction	167 (16.3)	4 (16.7)	121 (15.4)	2 (33.3)	40 (19.1)
Smoking History^b^ [# of patients (% of patients)]					
w/smoking history	533 (52.1)	12 (50.0)	443 (56.5)	5 (83.3)	73 (34.9)
Current Smoker^b^ [# of patients (% of patients)]					
Current smoker	109 (10.7)	5 (20.8)	95 (12.1)	0 (0.0)	9 (4.3)
**Prescribed Dose** (Gy[RBE=1.0 for photon and RBE=1.1 for proton])					
Median (range)	60 (60–81)	61.5 (60–70)	60 (60–81)	64.5 (60–70)	63 (60-74.4)
**Tumor within the oral cavity or oropharynx** [# of patients (% of patients)]					
Patients	690 (54.5)	26 (100)	503 (55.6)	8 (88.9)	153 (46.9)
MCR^c^ (# of patients)	1023	24	784	6	209
MCA^d^ (# of patients)	243	2	121	3	117
**Total** (# of patients)	1266	26	905	9	326

a Ctr, Control group.

b Data collected from Mayo Clinic Rochester only.

c MCR, Mayo Clinic Rochester.

d MCA, Mayo Clinic Arizona.

### Diagnosis and Staging of ORN

Patients with ORN were identified by experienced physicians clinically (bone exposure on physical examination), radiographically (Panorex, CT, MR, PET), and/or pathologically *via* debridement and resection/mandibulectomy. Patients with ORN were staged using the Marx system (27) based on their treatments with Trental/vitamin E, hyperbaric oxygen therapy (HBO), debridement, and mandibulectomy (**Supplemental Materials Section 1** for details of Marx staging). Details of demographic and graded Marx staging information for all ORN patients are listed in **Supplemental Table 1**.

### Treatment Plans and Dose/LET Calculation

Both VMAT and PBSPT plans were generated using a commercial treatment planning system, Eclipse™ (Varian medical system, Palo Alto, CA) based on patients’ simulation CTs (**Supplemental Materials Section 2** for details of treatment planning). All plans were evaluated to ensure that institutional dose volume constraints (DVCs) were met, if possible. The LET distributions of PBSPT plans were computed using an in-house fast Monte Carlo dose/LET calculation engine with a minimum electron energy cutoff of 50 keV (28, 29).

### Case-Matching of VMAT and PBSPT Patients

To minimize the impact of the possible imbalance in clinical factors between VMAT and PBSPT patients in the dosimetric comparison (dose and LET) between VMAT and PBSPT and the resulting RBE quantification, a subset of case-matched cohort of 335 patients from each modality was selected to carry out the analysis. The VMAT-PBSPT matched cohort was based on balancing clinical factors including gender, tumor stage, concurrent chemotherapy, hypertension, diabetes, dental extraction, current smoker, and smoking history using the greedy nearest neighbor matching of propensity scores. The propensity score represented the probability of one patient being treated with VMAT or PBSPT based on the observed clinical factors. In the greedy nearest neighbor matching, the VMAT patient whose propensity score was the closest to that of the PBSPT patient was selected as the match without replacement (30). The matched cohort was generated using “MatchIt” package of R (version 4.1.2).

### Dose Volume Constraints (DVCs)

Dose volume histograms (DVHs) were calculated within Eclipse™. DVH indices of absolute volumes receiving doses of at least 40 Gy[RBE] (V40Gy[RBE](cc)), 50 Gy[RBE] (V50Gy[RBE](cc)), 60 Gy[RBE] (V60Gy[RBE](cc)), 70 Gy[RBE] (V70Gy[RBE](cc)), and 75 Gy[RBE] (V75Gy[RBE](cc)) to the mandible, the minimum dose irradiated to the hottest 0.01 cc of mandible (D0.01cc), and mean dose (Dmean) of mandible were extracted for analysis.

The distribution of DVH indices were visualized using box plots. The bottom and top of each box were 25 and 75 percentiles from the population, and the middle line indicated the median value. Minimum and maximum values of whiskers were set as half of the interquartile range below or above the 25 or 75 percentiles.

The performance of all DVH indices was evaluated by calculating the area under curve (AUC) of the corresponding receiver operating characteristic (ROC) curve. Critical volumes were determined as the thresholds that performed at the optimal operating point of ROCs by minimizing the distance of the optimal operating point to the ideal case [true positive rate (TPR) = 1, false positive rate (FPR) = 0]. To estimate the uncertainties in the derived critical volumes and the ROC curve, we performed a bootstrapping of 1,000 times. 95% bootstrap confidence intervals of AUCs and critical volumes were computed. The true positive rates and false positive rates for the critical volumes were also calculated. Using this data we created volume tolerance curves of the mandible for both VMAT and PBSPT. This represented the possible tolerance volumes at different doses from both modalities.

### Calculation of Empirical RBEs

We derived empirical proton RBEs by comparing volume tolerance curves between VMAT and PBSPT. According to the definition of RBE, it is the ratio of doses to reach the same clinical endpoint when a novel RT modality, such as PBSPT, is compared to photon irradiation (in this case VMAT). In this study, we considered the critical volumes of VMAT and PBSPT as the endpoints. We sought the equivalent constraint doses based on the VMAT volume tolerance curve *via* linear interpolation so the critical volumes of VMAT were equal to those of PBSPT at different physical doses. Empirical RBEs of PBSPT for mandible ORN were obtained by calculating the ratio between the derived equivalent constraint doses and physical doses (RBE=1.0) of PBSPT. 95% confidence intervals of RBEs were obtained using a bootstrapping of 1,000 times.

### Dose LET Volume Histograms (DLVHs)

**Figure 1:**
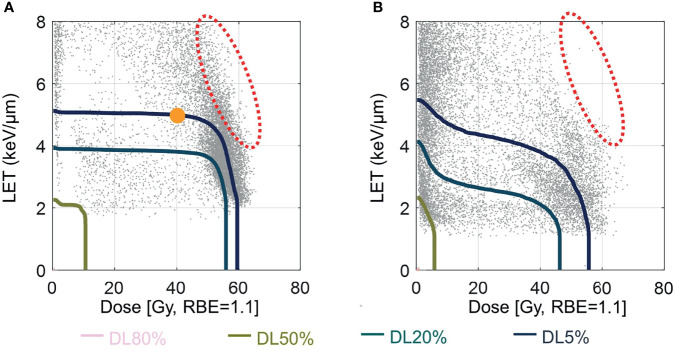
Dose-LET volume histogram (DLVH) of mandible. **(A)** DLVHs of mandible for a typical patient with ORN and **(B)** without ORN. DL5%, DL20%, and DL50% lines were plotted with different colors. Red dashed ovals indicate an area containing voxels with moderate doses but high LETs for this ORN patient.

DLVH is a recently proposed cumulative volume histogram tool following the similar statistical study concept of DVH and aimed to evaluate the impact of LET by bypassing the uncertainties in the existing RBE models (25). It presents dosimetric variables including dose, LET, and normalized volume, all of which can be calculated relatively accurately. **Figure 1** **A** illustrates a typical DLVH plot, in which the X axis is the RBE=1.1 dose and the Y axis is LET. Details of DLVH are included in **Supplemental Materials Section 3**.

### Statistical Analysis

All DVH indices were tested using Mann-Whitney U test. Categorical factors (gender, stage, chemotherapy, hypertension, diabetes, dental extraction, smoking history, and current smoker) of patients were tested using χ^2^ test and numerical factors (age and prescription doses) were tested using two-sided two-sample *t* test. 95% confidence intervals of critical volumes, AUCs, equivalent constraint doses, and RBEs were derived based on a bootstrapping of 1000 times. All the statistical analyses were scripted using Matlab 2019a (MathWorks, Inc., Natick, Massachusetts, United States). *P*-value smaller than 0.05 was considered statistically significant.

## Result

### Patient Characteristics

Of 1,266 patients included in this study, the median age when patients finished the treatment was 62 (interquartile range: 15) years, and 327 (25.8%) were women. Of the VMAT group, 26 (2.8%) patients developed ORN of the mandible (grade≥2, Common Terminology Criteria for Adverse Events, CTCAE v4.0), while 9 (2.7%) patients experienced ORN of the mandible (grade≥2, CTCAE v4.0) in the PBSPT group. These 35 patients constituted the ORN group, while all the others constituted the control group. The ORN incidence rates were 4.91% (VMAT) and 4.97% (PBSPT) respectively among those oral cavity and oropharynx patients (**Table 1**).

No statistically significant differences of age (*p*=0.468), gender (*p*=0.424), tumor stage (*p*=0.797), prescription dose (*p*=0.349), concurrent chemotherapy (*p*=0.175), conditions of hypertension (*p*=0.987), diabetes (*p*=0.227), dental extraction (*p*=0.582), smoking history (*p*=0.611), or current smoker (*p*=0.245) between the ORN and control group were observed in the overall patient cohort (**Supplemental Table 2** column 2).

For the clinical factor comparison of the ORN vs. control group in the patients either treated with VMAT or PBSPT, no statistically significant associations of age, gender, tumor stage, prescription dose, chemotherapy, hypertension, diabetes, dental extraction, or current smoker to ORN were observed (**Supplemental Table 2** column 3 and 4). Tests showed that smoking history was significantly associated with the ORN occurrence in patients treated with PBSPT only (*p*=0.015), but not in patients treated with VMAT (*p*=0.527). Detailed analysis regarding these risk factors to the ORN incidence will be reported in a separate study (31).

For the clinical factor comparison of the patients treated with PBSPT vs. VMAT in either the ORN group or the control group, no aforementioned clinical factors were found to be significantly different in the ORN group (**Supplemental Table 3** column 3). Tumor stage, hypertension, and smoking history were observed to be significantly different between VMAT and PBSPT in the control group (*p*<0.05) (**Supplemental Table 3** column 4) and thus the overall patient cohort (**Supplemental Table 3** column 2).

### Characteristics of Case-Matching Patient Cohort

For the case-matched patient subset cohort between the two modalities, all of 335 PBSPT patients were included in this subset cohort, including 9 ORN patients. In the case-matched photon group of 335 patients, 8 patients experienced osteoradionecrosis. After the case matching, all clinical factors were balanced between VMAT and PBSPT in both the ORN and control groups (*p*>0.05, **Supplemental Table 4**). Similar to the results based on the entire patient cohort, no clinical factors showed significant difference between the ORN and control group for both modalities, except smoking history between the ORN and control group in PBSPT patients (*p*=0.046) (**Supplemental Table 5**). All the dosimetric analysis below were based on this case-matched patient subset cohort.

### V40Gy[RBE], V50Gy[RBE], and V60Gy[RBE] Were Statistically Significantly Different (*p*<0.05) Between the ORN Group and Control Group for Both VMAT and PBSPT in All Comparisons

For all patients, and patients in each individual modality, V40Gy[RBE], V50Gy[RBE], and V60Gy[RBE] were statistically significantly different between the ORN group and control group in all comparisons (V40Gy[RBE]: *p*=0.007 and *p*=0.004; V50Gy[RBE]: *p*=0.004 and *p*=0.002; V60Gy[RBE]: *p*=0.014 and *p*=0.003 [VMAT and PBSPT], **Table 2**). The DVH indices of V70Gy[RBE], V75Gy[RBE], Dmean, and D0.01cc are not considered in the following analysis since they are not statistically significantly different in some or all comparisons.

**Table 2:** *P*-values between ORN and control patients for overall, VMAT and PBSPT patients in the case matched patient cohort (n=670).

Metrics	Overall	Photon	Proton
V40Gy[RBE=1.0 for photon and RBE=1.1 for proton](cc)	<0.001	0.007	0.004
V50Gy[RBE=1.0 for photon and RBE=1.1 for proton](cc)	<0.001	0.004	0.002
V60Gy[RBE=1.0 for photon and RBE=1.1 for proton](cc)	<0.001	0.014	0.003
V70Gy[RBE=1.0 for photon and RBE=1.1 for proton](cc)	0.011	0.057	0.087
V75Gy[RBE=1.0 for photon and RBE=1.1 for proton](cc)	0.066	0.110	0.322
D0.01cc(Gy[RBE=1.0 for photon and RBE=1.1 for proton])	0.015	0.232	0.041
Dmean(Gy[RBE=1.0 for photon and RBE=1.1 for proton])	0.008	0.003	0.020

**Figure 2:**
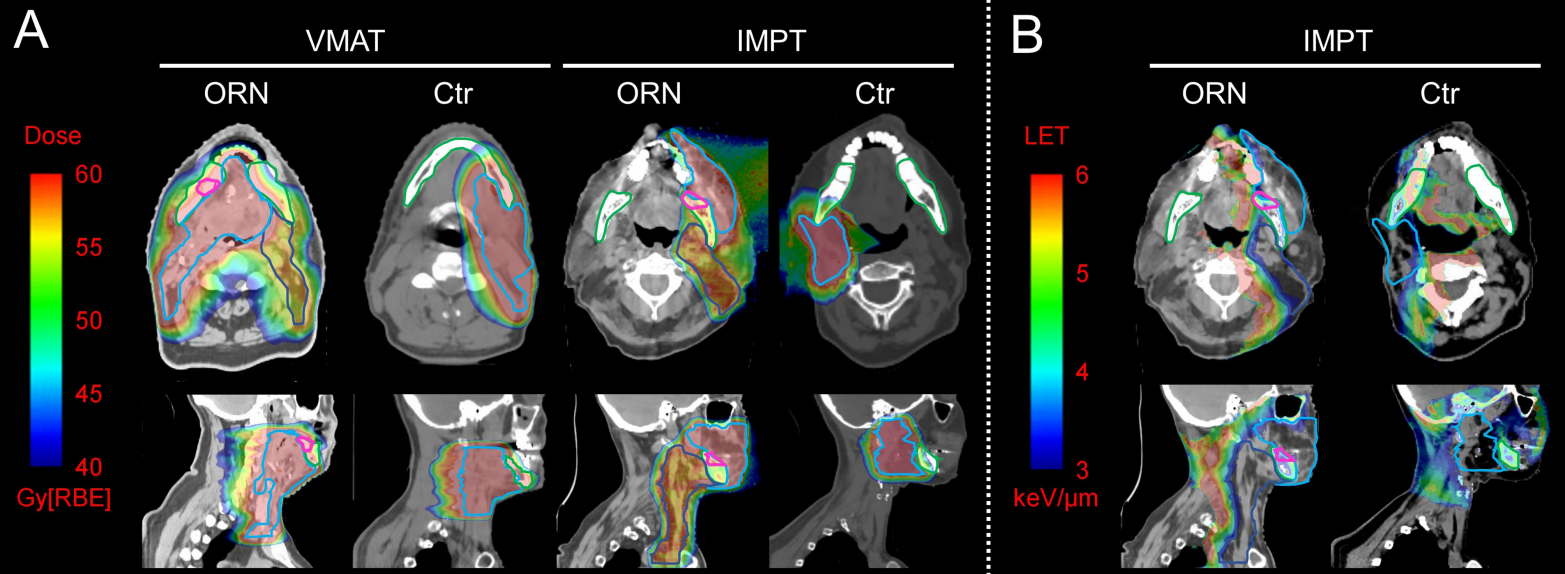
Dose and LET distributions of representative patients with and without osteoradionecrosis (ORN) treated with volumetric-modulated arc therapy (VMAT) and pencil-beam-scanning proton therapy (PBSPT) **(A)** Axial (*top* row) and sagittal (*bottom* row) view of dose distributions of typical patients treated with VMAT (*left* two columns) and PBSPT (*right* two columns) with (1^st^ and 3^rd^ column) and without (2^nd^ and 4^th^ column) ORN. **(B)** Axial (*top* row) and sagittal (*bottom* row) view of LET distributions of typical patients treated with PBSPT with (*left* column) and without (*right* column) ORN. Contour colors: pink: ORN injury regions; cyan: CTV_high_; blue: CTV_low_; green: mandible.

**Figure 2** **A** shows the axial (*top* rows) and sagittal view (*bottom* rows) of the dose distributions from typical patients with (1^st^ and 3^rd^ column) and without ORN (2^nd^ and 4^th^ column) from VMAT (*left* two columns) and PBSPT (*right* two columns) (one patient for each modality). The ORN region is contoured in pink. Note that these injury regions of patients treated with PBSPT were typically located distal to the edge of the high-risk CTV (CTV_High_, cyan) in the beam direction.

### Critical Volumes of PBSPT Are Lower Than Those of VMAT

**Figure 3:**
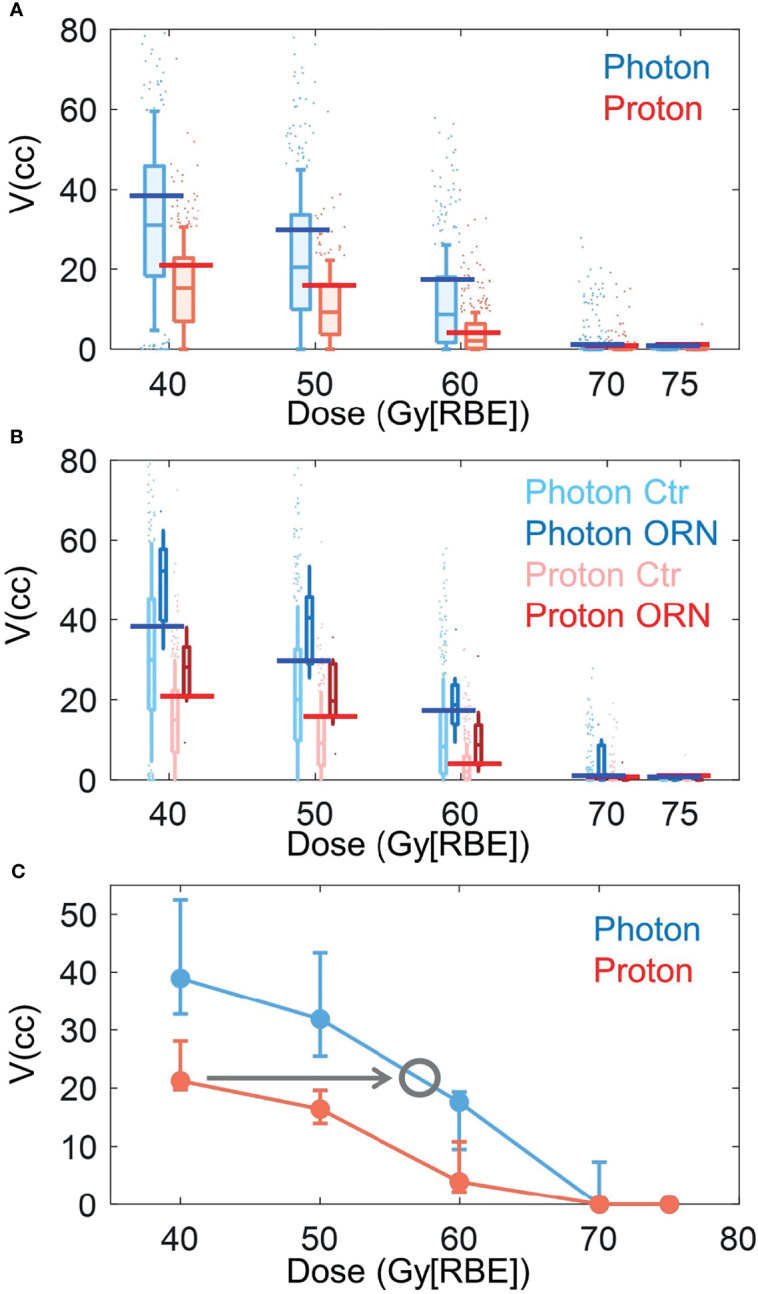
Box plots of volume value distributions of DVH indices. **(A)** Box plots of volume value distributions of V40Gy[RBE], V50Gy[RBE], V60Gy[RBE], V70Gy[RBE], and V75Gy[RBE] for all photon (blue) and proton (red) patients. **(B)** Box plots of volume value distributions of V40Gy[RBE], V50Gy[RBE], V60Gy[RBE], V70Gy[RBE], and V75Gy[RBE] for patients with (dark color) and without (light color) ORN treated with VMAT (blue) and PBSPT (red). Blue and red horizontal lines in **(A)** and **(B)** indicated the derived DVCs for VMAT and PBSPT, respectively. **(C)** Volume tolerance curves for VMAT (blue) and PBSPT (red) based on the derived DVCs. Gray circle indicates the position at the intersection between the VMAT volume tolerance curve and a horizontal line (indicated by the gray arrow) with the same critical volume value as the corresponding DVC of the PBSPT volume tolerance curve. The corresponding dose at the gray circle was the equivalent constraint dose. Error bars indicate the 95% confidence intervals obtained by a bootstrapping of 1,000 times.

**Figure 3** shows the box plots of V40Gy[RBE], V50Gy[RBE], V60Gy[RBE], V70Gy[RBE] and V75Gy[RBE] between the patients treated with VMAT and PBSPT overall (**Figure 3** **A**) and between the ORN and control group in VMAT and PBSPT, respectively (**Figure 3** **B**). PBSPT showed better sparing of the mandible than VMAT as suggested by lower dose volumes of V40Gy[RBE], V50Gy[RBE], and V60Gy[RBE] (V40Gy[RBE]: 30.94 cc vs. 15.25 cc; V50Gy[RBE]: 20.44 cc vs. 9.25 cc; V60Gy[RBE]: 8.68 cc vs. 2.12 cc [VMAT vs PBSPT, medians]) (**Figure 3** **A**). Despite having improved sparing of the mandible in PBSPT compared to VMAT, the proton group did not show obvious reduction in the incidence rate of ORN. The critical volumes of the derived mandible DVCs in PBSPT were lower than those of the derived mandible DVCs in VMAT for V40Gy[RBE], V50Gy[RBE], and V60Gy[RBE] (VMAT: V40Gy[RBE]: 38.96 cc (95% CI: 32.74-52.49 cc); V50Gy[RBE]: 31.85 cc (95% CI: 25.49-43.39 cc); V60Gy[RBE]: 17.61 cc (95% CI: 9.49-19.35 cc); PBSPT: V40Gy[RBE]: 21.26 cc (95% CI: 19.75-29.44 cc); V50Gy[RBE]: 16.42 cc (95% CI: 13.95-19.63 cc); V60Gy[RBE]: 3.95 cc (95% CI: 2.19-10.82 cc)) (**Figure 3** **C** and **Table 3**). High AUCs, high TPRs, and low FPRs of V40Gy[RBE], V50Gy[RBE], and V60Gy[RBE] from the derived DVCs were observed (**Table 3**).

**Table 3:** Critical volumes, AUC, true positive rate (TPR) and false positive rate (FPR), equivalent constraint dose in VMAT and empirical RBE based on derived DVCs for VMAT and PBSPT.

		V40Gy[RBE](cc)*	V50Gy[RBE](cc)*	V60Gy[RBE](cc)*
**Critical volumes (cc) (95%CI)**				
Photon		38.96 (32.74-52.49)	31.85 (25.49-43.39)	17.61 (9.49-19.35)
Proton		21.26 (19.75-29.44)	16.42 (13.95-19.63)	3.95 (2.19-10.82)
**AUC (95%CI)**				
Photon		0.766 (0.690-0.863)	0.789 (0.719-0.866)	0.799 (0.665-0.841)
Proton		0.784 (0.662-0.883)	0.808 (0.695-0.905)	0.795 (0.702-0.884)
**Performance**				
Photon	TPR	0.875	0.750	0.750
	FPR	0.343	0.256	0.245
Proton	TPR	0.778	0.778	0.778
	FPR	0.273	0.224	0.331
**Equivalent constraint dose in photon (Gy[RBE=1.0]) (95%CI)**		57.44 (48.59-59.51)	60.67 (55.67-63.55)	67.75 (62.67-68.76)
**Empirical RBE (95%CI)**		1.580 (1.336-1.636)	1.335 (1.225-1.398)	1.242 (1.149-1.261)

*RBE=1.0 for photon and RBE=1.1 for proton.

**Figure 2** **B** shows the axial (*top* rows) and sagittal view (*bottom* rows) of the LET distributions from typical patients with (*left*) and without ORN (*right*) treated with PBSPT. Elevated LET distribution appears to correlate with the injury sites with moderate doses (above 40 Gy[RBE=1.1]).

### Equivalent Constraint Doses and Empirical RBEs

Empirical RBEs were calculated by comparing the volume tolerance curves between PBSPT and VMAT (**Figure 3** **C**) *via* equivalent constraint dose analysis. Empirical RBEs of 1.58 (95% CI: 1.34-1.64), 1.34 (95% CI: 1.23-1.40), and 1.24 (95% CI: 1.15-1.26) were obtained at a proton dose of 40 Gy[RBE=1.1], 50 Gy[RBE=1.1], and 60 Gy[RBE=1.1], respectively (**Table 3**). The empirical RBEs decreased with the increase of the physical dose in PBSPT.

### DLVHs of PBSPT Showed that the LET Top Edges at Each Dose Bin Were Decreased With the Increase of the Physical Dose

**Figures 1A, B** show DLVHs of typical PBSPT patients with and without mandible ORN, respectively. More voxels of high LETs above 5 keV/µm were observed in the ORN patients (above a dose of 40 Gy[RBE=1.1]) than those in the control patients (comparing **Figure 1** **A** to **Figure 1** **B**). Both DLVHs showed a decrease of the LET top edges at each dose bin when the physical dose increased from 40 to 60 Gy[RBE=1.1]. However, a generally higher LET distribution was observed in the ORN patients than that in the control patients (~8 keV/µm to ~4 keV/µm in the ORN group and ~6 keV/µm to ~3 keV/µm in the control group when the physical dose was increased from 40 to 60 Gy[RBE=1.1], as shown by comparing red ovals in **Figures 1A, B**).

## Discussion

We included 1,266 patients at our institution in order to report the incidence rate of mandible ORN and all known clinical factors possibly related to mandible ORN for both modalities. Thus, it would give the readers a more comprehensive picture of the incidence of mandible ORN in H&N cancer patients treated with both VMAT and PBSPT and its associated clinical factors, which are clinically meaningful. As far as we know, our work presents one of the largest and most comprehensive retrospective adverse event studies focusing on mandible ORN for H&N cancer patients treated with VMAT and PBSPT, particularly PBSPT (335 patients).

The major purpose of this work was to study the empirical RBE for mandible ORN in head and neck cancer patients treated with PBSPT. It is thus important to balance VMAT and PBSPT patient groups in all known clinical factors possibly associated with mandible ORN to mitigate the influence of these confounding clinical factors and bias. As such, we used the method of the greedy nearest neighbor matching of propensity scores to match all known clinical factors to balance the two patient groups (335 patients from the VMAT group and 335 patients from the PBSPT group, in total 670 patients) and perform the case-matched cohort study to investigate the dosimetric effects (i.e., empirical RBE induced by higher LET in PBSPT) in the incidence of mandible ORN in H&N cancer patients treated with PBSPT.

No ORN-related clinical factors were found to be significantly different in the ORN group comparing patients treated with PBSPT and VMAT (**Supplemental Table 3**). However, tumor stage, hypertension, and smoking condition were observed to be significantly different between VMAT and PBSPT in the control group (*p*<0.05). The unmatched patients in terms of these three factors in the control groups may introduce the bias in the dosimetric comparison between VMAT and PBSPT and the resulting RBE quantification. We therefore formed a 1:1 case-matched cohort (335 patients treated with both PBSPT and VMAT including both the ORN and control groups). We performed all the remaining studies based on this case-matched patient subset cohort.

In this study, we investigated the empirical RBEs of PBSPT for mandible ORN based on patient outcomes. DVCs with moderate dose indices (V40Gy[RBE], V50Gy[RBE], and V60Gy[RBE]), instead of the DVCs with high dose indices (V70Gy[RBE] and V75Gy[RBE]), were found to have statistically significant differences between the ORN group and control group for both VMAT and PBSPT (**Table 2**). For VMAT, this dose volume effect at moderate doses has been reported in multiple studies related to ORN (32–37). The derived DVCs for VMAT are consistent with the reported results (**Table 3**).

DVCs for mandible ORN in PBSPT were first reported in this study. The mandible DVCs such as V40Gy[RBE], V50Gy[RBE], and V60Gy[RBE] derived for VMAT and PBSPT were good predictors of the possible incidence of mandible ORN as demonstrated by the high AUCs, high TPRs, and low FPRs in **Table 3**. The derived DVCs may serve in the future as clinical guidance for plan evaluation and optimization to minimize the risk of ORN in PBSPT.

DVH indices showed PBSPT patients received much less dose at the mandible than VMAT patients (**Figure 3** **A**). This suggested that PBSPT had significantly better dose sparing of the mandible than VMAT. However, a comparable incidence rate of ORN was observed between PBSPT and VMAT. This could be multifactorial, such as smaller sample size in PBSPT, patient heterogeneity, and preexisting dental issues/hardware interactions etc. After case-matching, our results suggested that PBSPT patients had lower critical tolerance volumes than those in VMAT patients (**Figure 3** **B** and **Table 3**). Thus, RBEs for grade≥2 mandible ORN between 40 and 60 Gy[RBE=1.1] may be higher than 1.1. The significant underestimation of RBE in moderate dose regions may lead to unexpected mandible ORN in H&N cancer treated with PBSPT.

We theorized that the increased LET of PBSPT results in RBEs larger than 1.1. This can be supported by the fact that the injury sites of ORN in PBSPT occurred at regions distal to the edges of the CTVs in the beam direction, which usually coincide with the position of the Bragg peak with high LET radiation (**Figure 2** **B**). We observed a decrease of empirical RBE in relation to the increase of physical dose from 40 Gy[RBE=1.1] to 60 Gy[RBE=1.1] (**Table 3**). This could be explained by the DLVH plots, in which the top edges of LET at each dose bin were decreased with the increase of the proton physical dose (**Figure 1**). In the future it may be possible to reduce the incidence of ORN by LET-guided robust optimization to achieve more desirable LET distributions in PBSPT (7, 14). However, the root causes of the RBE of >1.1 and its relation to the potential LET-enhancing effects needs to be further elucidated.

This study has certain limitations. Although a large patient cohort (1,266 patients) was included in this study, the number of patients with ORN (35) was small. Considering the uncertainties in RT treatment planning and delivery, the RBEs derived in this study can only be considered as rough estimates. Investigations combining data from multiple institutions are needed to verify our conclusions and derive more accurate RBE models. We are trying to establish research collaborations with more proton centers to share patient outcomes data. We hope that we can collect far more patient data with mandible ORN from multiple institutions and this limitation can be resolved with efficient and secure data sharing enabled by advanced algorithms such as blockchain (38). RBE also varies by clinical endpoints and tissue types. In this study, we only investigated mandible-specific empirical RBEs for ORN. With ORN being a late occurring complication, a study with a sufficiently long follow-up time may provide more accurate results.

Complicated mechanisms can also be involved in the ORN development, such as radiation-induced small vessel obliteration compromising the blood supply to the mandible, radiation-induced death of osteoblasts, etc. (39). Local dose effect to blood vessels could be enhanced by proximity to high density bone with increased LET (40). We did not consider which functioning units of the mandible were damaged to induce ORN either. In order to further reveal the underlying mechanisms of LET-enhancing effects in PBSPT, voxel-based analysis within the injury regions would be helpful.

In conclusion, V40Gy[RBE], V50Gy[RBE], and V60Gy[RBE] were found to have statistically significant differences between the ORN group and control group for both VMAT and PBSPT, which formed the volume tolerance curves. The critical volumes of the DVCs were higher in VMAT than PBSPT, suggesting LET-enhancing effects in PBSPT. *Via* equivalent constraint dose analysis based on the volume tolerance curves, we obtained empirical RBEs, which decreased with the increase of proton physical doses. This could be explained by DLVH plots, in which the LET top edges at each dose bin decreased with the increase of proton physical doses. Our study suggested a RBE substantially larger than 1.1 at moderate doses (between 40 and 60 Gy[RBE=1.1]) with high LET. Reducing the LET at moderate doses may minimize the incidence of ORN for H&N cancer treated with PBSPT.

## Data Availability Statement

The raw data supporting the conclusions of this article will be made available upon request to the corresponding authors.

## Ethics Statement

The studies involving human participants were reviewed and approved by Mayo Clinic Institutional Review Board 13-005709. Written informed consent from the participants’ legal guardian/next of kin was not required to participate in this study in accordance with the national legislation and the institutional requirements.

## Author Contributions

YY designed and conducted the analysis. OM, MH, WW, LM, J-CR, SSc, SP, and RF clinically identified and confirmed the patient cohort. SSh, AA, MF, and JA collected and aggregated the patient data. YY cleaned the data and carried out the statistical tests. YY and WL reviewed the statistical analysis. MB, MF, SP, and RF discussed results. YY and WL wrote the manuscript. RF and WL conceived and supervised the project. All authors read, revised, and approved the final manuscript.

## Funding

This research was supported by Arizona Biomedical Research Commission Investigator Award (ADHS16-162521), the Lawrence W. and Marilyn W. Matteson Fund for Cancer Research, and the Kemper Marley Foundation.

## Conflict of Interest

WL reports grants from NIH/NCI, outside the submitted work; in addition, WL has a pending US patent: “An Accurate and Efficient Hybrid Method Based on Ray Casting to Calculate Physical Dose and Linear Energy Transfer (LET) Distribution for Intensity-modulated Proton Therapy”, which is licensed to.decimal LLC. SSc reports personal fees from uptodate/editor and author, outside the submitted work. RF reports royalties from Elsevier, textbook editing, and sales of the book; royalties from uptodate/editor and author; unrestricted research funding from Hitachi for the named professorship and royalties from Bionix for sales of TruGuard from the sold patent. All listed above are outside the submitted work.

The remaining authors declare that the research was conducted in the absence of any commercial or financial relationships that could be construed as a potential conflict of interest.

## Publisher’s Note

All claims expressed in this article are solely those of the authors and do not necessarily represent those of their affiliated organizations, or those of the publisher, the editors and the reviewers. Any product that may be evaluated in this article, or claim that may be made by its manufacturer, is not guaranteed or endorsed by the publisher.
