# Supplementary material for: Empirical Relative Biological Effectiveness (RBE) for Mandible Osteoradionecrosis (ORN) in Head and Neck Cancer Patients Treated With Pencil-Beam-Scanning Proton Therapy (PBSPT): A Retrospective, Case-Matched Cohort Study
Source: Front Oncol. 2022 Mar 3;12:843175. doi: 10.3389/fonc.2022.843175 (PMC8928456; doi:10.3389/fonc.2022.843175)
Supplement: Supplementary file 1 [file DataSheet_1.docx]

**Supplemental Materials Section 1: Diagnosis and staging of ORN**

Patients with ORN were identified by experienced physicians clinically (bone exposure on physical examination), radiographically (Panorex, CT, MR, PET), and pathologically via debridement and resection/mandibulectomy with fibula free flap reconstruction. Patients with ORN were staged using the Marx system(1) based on their treatments with Trental/vitamin E, hyperbaric oxygen therapy (HBO), debridement, and mandibulectomy: Stage I-exposed alveolar bone with -no pathologic fracture, which responds to HBO. Stage II-no response to HBO, requiring sequestrectomy and saucerization. Stage III-full thickness bone damage or pathologic fracture requiring complete resection and reconstruction with free tissue. Details of demographic and graded Marx staging information for all ORN patients are listed in Supplemental Table 1.

**Supplemental Materials Section 2: Treatment plans**

Both VMAT and PBSPT plans were generated using a commercial treatment planning system (TPS), Eclipse^TM^ (Varian medical system, Palo Alto, CA) based on patients’ simulation CTs. For VMAT, planning target volumes (PTV), 3-mm margin expansion of clinical target volumes (CTVs), were used for plan optimization. The photon optimizer (PO) model in Eclipse^TM^ was used for VMAT treatment planning, and the analytical anisotropic algorithm (AAA) was used for dose calculation. Typically, 2 to 3 arcs were used for each plan. For PBSPT, single-field optimization (SFO) or multi-field optimization (MFO) (2) based planning was employed to achieve clinically acceptable proton plans. Pencil beam convolution superposition (PCS) optimization algorithm was used for SFO and the nonlinear uniform proton optimizer (NUPO) robust optimization was employed for MFO.

**Supplemental Materials Section 3: Dose LET volume histograms (DLVHs)**

DLVH is a recently proposed cumulative volume histogram tool following the similar statistical study concept of DVH and aimed to evaluate a plan by bypassing the uncertainties in the existing RBE models(3). It presents dosimetric variables including dose, LET, and normalized volume, all of which can be calculated relatively accurately. Figure 1a illustrates a typical DLVH plot, in which the X axis is the RBE=1.1 dose and the Y axis is LET. The contour lines correspond to different percentage volume of the selected structure. Similar to DVH indices such as D*v*%(*d*), *i.e.*, the *v* percentage volume of a structure has a dose of at least *d* Gy[RBE=1.1], these iso-volume lines, DL*v*%(*d*,*l*), indicate the *v* percentage volume of a structure that has a dose of at least *d* Gy[RBE=1.1] and an LET of at least *l* keV/µm. For example, the orange point in Figure 1a indicates that 5% volume of the structure has a dose of at least 40 Gy[RBE=1.1] and an LET of at least 5.0 keV/µm. Voxels were mapped onto DLVH as gray dots according to their corresponding dose and LET.

**Supplemental Table 1** Demographic information and Marx stages of ORN patients

| Patient # | Sex | Age | Stage | RT Type | Prescribed Dose (cGy[RBE])* | Institute^a^ | Marx Stage |
| --- | --- | --- | --- | --- | --- | --- | --- |
| 1 | M | 57 | Stage III | VMAT | 6000 | MCR | 3 |
| 2 | M | 49 | Stage II | VMAT | 6000 | MCR | 2 |
| 3 | F | 57 | Stage IVA | VMAT | 7000 | MCR | 3 |
| 4 | M | 53 | Stage IVA | VMAT | 6000 | MCR | 3 |
| 5 | F | 69 | Stage IVB | VMAT | 7000 | MCR | 3 |
| 6 | F | 63 | Stage X | VMAT | 6300 | MCR | 1 |
| 7 | M | 52 | Stage IVA | VMAT | 7000 | MCR | 3 |
| 8 | M | 77 | Stage IVA | VMAT | 6000 | MCR | 1-right, 3-left |
| 9 | M | 46 | Stage IVA | VMAT | 6000 | MCR | 1 |
| 10 | F | 53 | Stage I | VMAT | 6000 | MCR | 1 |
| 11 | M | 51 | Stage IVA | VMAT | 7000 | MCR | 1 |
| 12 | M | 63 | Stage IVA | VMAT | 7000 | MCR | 1 |
| 13 | M | 57 | Stage IVC | VMAT | 7000 | MCR | 3 |
| 14 | M | 56 | Stage III | VMAT | 7000 | MCR | 3 |
| 15 | M | 68 | Stage IVA | VMAT | 6000 | MCR | 1 |
| 16 | M | 67 | Stage IVA | VMAT | 6000 | MCR | 1 |
| 17 | F | 56 | Stage I | VMAT | 6000 | MCR | 3 |
| 18 | F | 59 | Stage IVA | VMAT | 7000 | MCR | 2-right, 2-left |
| 19 | M | 69 | Stage IVA | VMAT | 6000 | MCR | 2-right, 2-left |
| 20 | M | 50 | Stage II | VMAT | 6000 | MCR | 2 |
| 21 | M | 63 | Stage III | VMAT | 6000 | MCR | 2 |
| 22 | M | 73 | Stage IVA | VMAT | 6996 | MCR | 3 |
| 23 | M | 64 | Stage IVA | VMAT | 6000 | MCR | 2 |
| 24 | M | 51 | Stage III | VMAT | 7000 | MCR | 2 |
| 25 | M | 59 | Stage X | VMAT | 7000 | MCA | 3 |
| 26 | M | 66 | Stage IVA | VMAT | 7000 | MCA | 3 |
| 27 | M | 46 | Stage IVA | PBSPT | 6450 | MCR | 1 |
| 28 | M | 60 | Stage IVA | PBSPT | 6996 | MCR | 1 |
| 29 | F | 46 | Stage IVA | PBSPT | 6000 | MCR | 1 |
| 30 | M | 75 | Stage IVA | PBSPT | 7000 | MCR | 2 |
| 31 | M | 68 | Stage I | PBSPT | 6000 | MCR | 3 |
| 32 | M | 58 | Stage X | PBSPT | 6000 | MCR | 2 |
| 33 | M | 83 | Stage III | PBSPT | 6000 | MCA | 1 |
| 34 | M | 74 | Stage III | PBSPT | 7000 | MCA | 1 |
| 35 | M | 59 | Stage I | PBSPT | 7000 | MCA | 1 |

^a^MCR: Mayo Clinic Rochester; MCA: Mayo Clinic Arizona
*RBE=1.0 for photon and RBE=1.1 for proton

**Supplemental Table 2** *P*-values between osteoradionecrosis and control patients of demographic characteristics for overall, VMAT and PBSPT patients in the consecutive cohort (n=1,266)

| Patient demographic information | Overall | Photon | Proton |
| --- | --- | --- | --- |
| Age | 0.468 | 0.250 | 0.740 |
| Gender | 0.424 | 0.660 | 0.391 |
| Tumor stage | 0.797 | 0.978 | 0.594 |
| Concurrent Chemotherapy^a^ | 0.175 | 0.228 | 0.541 |
| Hypertension^a^ | 0.987 | 0.934 | 0.806 |
| Diabetes^a^ | 0.227 | 0.377 | 0.336 |
| Dental Extraction^a^ | 0.582 | 0.871 | 0.387 |
| Smoking History^a^ | 0.611 | 0.527 | 0.015^*^ |
| Current Smoker^a^ | 0.245 | 0.168 | 0.604 |
| Prescribed Dose (Gy[RBE=1.0 for photon and RBE=1.1 for proton]) | 0.349 | 0.434 | 0.604 |

^a^Data collected from Mayo Clinic Rochester only
^*^indicates *p*<0.05

**Supplemental Table 3** *P*-values between VMAT and PBSPT patients of demographic characteristics for overall, osteoradionecrosis and control patients in the consecutive patient cohort (n=1,266)

| Patient demographic information | Overall | ORN | Ctr |
| --- | --- | --- | --- |
| Age | 0.557 | 0.319 | 0.471 |
| Gender | 0.165 | 0.439 | 0.198 |
| Tumor stage | <0.05 | 0.638 | <0.05 |
| Concurrent Chemotherapy^a^ | 0.236 | 0.842 | 0.244 |
| Hypertension^a^ | <0.05 | 0.361 | <0.05 |
| Diabetes^a^ | 0.556 | 0.464 | 0.610 |
| Dental Extraction^a^ | 0.154 | 0.361 | 0.199 |
| Smoking History^a^ | <0.05 | 0.141 | <0.05 |
| Current Smoker^a^ | <0.05 | 0.221 | <0.05 |
| Prescribed Dose (Gy[RBE=1.0 for photon and RBE=1.1 for proton]) | 0.644 | 0.914 | 0.652 |

^a^Data collected from Mayo Clinic Rochester only
^*^indicates *p*<0.05

**Supplemental Table 4** *P*-values between VMAT and PBSPT patients of demographic characteristics for overall, osteoradionecrosis and control patients in the case-matched patient cohort (n=670)

| Patient demographic information | Overall | ORN | Ctr |
| --- | --- | --- | --- |
| Age | 0.995 | 0.630 | 0.952 |
| Gender | 0.470 | 0.453 | 0.538 |
| Tumor stage | 0.921 | 0.460 | 0.858 |
| Concurrent Chemotherapy^a^ | 0.899 | 0.505 | 0.825 |
| Hypertension^a^ | 0.996 | 1.000 | 0.995 |
| Diabetes^a^ | 0.592 | 1.000 | 0.594 |
| Dental Extraction^a^ | 0.606 | 0.505 | 0.519 |
| Smoking History^a^ | 0.702 | 0.221 | 0.858 |
| Current Smoker^a^ | 0.390 | 0.296 | 0.247 |
| Prescribed Dose (Gy[RBE=1.0 for photon and RBE=1.1 for proton]) | 0.590 | 0.862 | 0.569 |

^a^Data collected from Mayo Clinic Rochester only

**Supplemental Table 5** *P*-values between osteoradionecrosis and control patients of demographic characteristics for overall, VMAT and PBSPT patients in the case-matched patient cohort (n=670)

| Patient demographic information | Overall | Photon | Proton |
| --- | --- | --- | --- |
| Age | 0.908 | 0.801 | 0.740 |
| Gender | 0.524 | 0.980 | 0.391 |
| Tumor stage | 0.904 | 0.821 | 0.594 |
| Concurrent Chemotherapy^a^ | 0.141 | 0.142 | 0.541 |
| Hypertension^a^ | 0.844 | 0.848 | 0.957 |
| Diabetes^a^ | 0.307 | 0.481 | 0.611 |
| Dental Extraction^a^ | 0.831 | 0.836 | 0.670 |
| Smoking History^a^ | 0.052 | 0.608 | 0.046^*^ |
| Current Smoker^a^ | 0.555 | 0.066 | 0.859 |
| Prescribed Dose (Gy[RBE=1.0 for photon and RBE=1.1 for proton]) | 0.322 | 0.377 | 0.604 |

^a^Data collected from Mayo Clinic Rochester only
^*^indicates *p*<0.05

**References**

1. Chronopoulos A, Zarra T, Ehrenfeld M, et al. Osteoradionecrosis of the jaws: Definition, epidemiology, staging and clinical and radiological findings. A concise review. *Int Dent J* 2018;68:22-30.

2. Quan EM, Li X, Li Y, et al. A comprehensive comparison of imrt and vmat plan quality for prostate cancer treatment. *International journal of radiation oncology, biology, physics* 2012;83:1169-1178.

3. Yang Y, Vargas CE, Bhangoo RS, et al. Exploratory investigation of dose-linear energy transfer (let) volume histogram (dlvh) for adverse events study in intensity-modulated proton therapy *Int J Radiat Oncol Biol Phys* 2020.
